# Moving Through a Textual Space Autistically

**DOI:** 10.1007/s10912-023-09797-y

**Published:** 2023-05-03

**Authors:** Hanna Bertilsdotter Rosqvist, Anna Nygren, Sarinah O’Donoghue

**Affiliations:** 1 School of Social Sciences, Södertörn University, Alfred Nobels Allé 7, S-141 89 Huddinge, Sweden; 2 HDK-Valand—Academy of Art and Design, University of Gothenburg, Gothenburg, Sweden; 3 Department of English, School of Language, Literature, Music, and Visual Culture, University of Aberdeen, Aberdeen, Scotland AB24 3FX

**Keywords:** Neurodivergent reading, Collective writing, Autoethnography, Autism fiction, Autistic fiction

## Abstract

This article is an investigation of neurodivergent reading practices. It is a collectively written paper where the focus is as much on an autoethnographic exploration of our autistic readings of autism/autistic fiction as it is on the read texts themselves. The reading experiences described come primarily from Yoon Ha Lee’s *Dragon Pearl* (2019) and Dahlia Donovan’s *The Grasmere Cottage Mystery* (2018), which we experience as opposite each other in how they depict their neurodivergent characters and speak to us as autistic readers. Through the article, we describe a formation of neurodivergent (critical) collective readings of autism/autistic fiction. The article contributes to an academic and activistic discourse around neurodivergent reader responses and power relations between neurodivergent and neurotypical readers and authors.

## Introduction

Autistic people have been assumed to “lack the social understanding to contemplate fiction, preferring non-fiction” (Chapple, Williams, et al. 2021, 1). As a way to challenge this assumption, Chapple, Williams, and colleagues note in their study on autistic reading habits in contrast to non-autistic people that autistic participants did not show any preference between fiction and non-fiction. Stressing that “social benefits of narrative texts extend to autistic readers,” the autistic group “reported social learning outcomes from reading,” making reading a way of providing autistic people with “important social learning experiences” (Chapple, Williams, et al. 2021, 1). Additionally, neuromixed conversations about literature have been suggested as a way to overcome the Double Empathy Problem, as theorized by Milton (2012) (see Chapple, Davis, et al. 2021). Milton defines the Double Empathy Problem as:

A disjuncture in reciprocity between two differently disposed social actors which becomes more marked the wider the disjuncture in dispositional perceptions of the lifeworld—perceived as a breach in the “natural attitude” of what constitutes “social reality” for “non-autistic spectrum” people and yet an everyday and often traumatic experience for “autistic people.” (Milton 2012, 884).

From this perspective, communication between differently disposed social actors can be understood as cross-cultural communication and, more specifically in this context, as cross-neurotype communication (Hillary 2020) or what we will refer to here as *neuromixed conversations*. Working as a bridge between autistic and non-autistic groups, neuromixed conversations about literature may work as a possibility for people across neurological divides to achieve “an individualized view of one another to explore their nuanced differences” (Chapple, Davis, et al. 2021, 1). Chapple, Davis, and colleagues, thus, point to the need to reevaluate the assumption that autistic readers do not *understand* fiction and the possibility of fiction as a way of being in a neuromixed world and creating bonds between people (Chapple, Davis, et al. 2021). We agree with their critique of the idea of the autistic reader as not interested in fiction. However, we want to stress that ideas of fiction (such as ideas of what is good or comprehensible) and how to read fiction are dominated by what McDermott (2022, 55) has referred to as “the norms and conventions of neurotypicality,” or “neuroconventional” norms. Neuroconventional norms of fiction have an impact on autistic fiction authors’ ways of writing as well as autistic readers’ ways of reading (including ideas of autistic readers’ ways of reading as illustrated in research). In this paper, we want to suggest that reading fiction *autistically* might mean something other than reading it *neuroconventionally*. This article aims to explore these differences and displacements.

Theorizing the neurotypical gaze, McDermott (2022, 55) shows how the “neurotypical gaze works to naturalise neuroconventional ideas” of ways of relating to other people and, by this, “misses or altogether discounts the non-normative ways,” such as neurodivergent ways of relating. Similarly, the affair of *autistic ways of reading*—represented by non-autistic people and contrasted to neuroconventional (non-autistic) ways of reading—can be interpreted as workings of the neurotypical gaze. From a neurotypical gaze, autistic ways of reading are valued on the basis of possible neuroconventional social outcomes: of making autistic people interact in more neurotypical ways (to provide autistic people with “important social learning experiences”) and supporting cross-neurotype social encounters.

Williams (2020, 123) has argued, firstly, the impossibility for her as an autistic reader to separate herself and her “insider perspective” from her work, stressing that “autoethnographic creative writing offers a way to reflect on my inter-relation with the subject matter.” Secondly, she argues, the task for the autistic reader/writer is to give a voice “to those [autistic people more generally] who are often overlooked” (123). Through this voice, the autistic reader/writer can show “the breadth of what ‘autistic-ness’ can be,” pointing out that “autism is heterogeneous in its nature and autistic people are diverse in ways that popular conventions and stereotypes don’t often afford” (123).

Following McDermott and Williams in this paper, we will read from our insider perspectives. By this, we mean our autistic bodyminds. We use the term “bodymind” as understood by Margaret Price: “because mental and physical processes not only affect each other but also give rise to each other—that is because they tend to act as one, even though they are conventionally understood as two—it makes more sense to refer to them together, in a single term” (Price 2015, 269). We will explore the possibilities of neuroqueer reading practices to denaturalize neuroconventional ideas of ways of reading and writing. By this, we also want to contribute to a more diverse exploration of what reading and writing can be. Yergeau (2018) defines the neuroqueer as a potentiality, a deferral, and a constant pursuit of new meanings, interpretations, and ways of being that transcend the here and now. Autism is “always located, but not fixed or fixable: it is an ecology in movement, a vital force through which the neuroqueer might come to know” (Yergeau 2018, 181). Translate this to reading (and writing) practices, which are, by definition, constantly in pursuit of and constructing new meanings, and it is possible to develop a neuroqueer reading practice. Reading neuroqueerly means undoing this mind/body split and valuing the body as a site of meaning and cultural significance.

We write this article collectively, coming from different academic backgrounds—primarily literature and sociology but also creative writing and queer studies. We know each other primarily through text. We have met physically but developed a relationship through writing together. This means our writing is a way of *being together*, both academically and socially. We often feel that our positions as autistic autism researchers are complicated ones (cf. Botha 2021a). They involve navigating social-academic conventions and traditions while at the same time managing our feelings about being objects of autism studies rather than the subjects and researchers, even when we are invited to share our perspectives. This collective writing process has become a room of our own where we can develop and deepen our thoughts without the need to constantly adapt them to a neurotypical reader. Throughout, we mingle our collective autoethnographic accounts with research accounts and theories to illustrate the work with the text as thinking about autism with each other in itself. We have chosen to refer to our autoethnographic voices in the text with a collective *One of us*. This is a way of stressing the text as written in a collective space, the collective *I* as *One of us*. *One of us* is also used as an expression of a “joint action,” which feminist researchers Francis and Hey have stressed as a “core to feminist action over the years” but in particular within academia, where joint action counter-narrates the position as “individual experts” (Francis and Hey 2009, 231). The use of a collective *I* is a way to counter-narrate the image of “the sole,” individualized neurodivergent and, instead, stress the presence of a neurodivergent togetherness; it is also a way to protect ourselves against structural violence and position ourselves—as neurological minority (stigmatized) selves.

In our readings, we will use quotes from our reading of journals and our conversations with each other (referred to as *One of us*) to find these tools and strategies for our autistic (and collective) reader moves. By this, we do not acknowledge a hierarchical order of source material, academic sources, and personal notes. The novels we will refer to throughout the text have been chosen through extensive reading of autism/autistic fiction to find aspects that interest us as readers—an interest that is sometimes critical, sometimes embracing. The novels have been chosen primarily out of interest, although this interest takes on different forms: One kind of interest is the euphoric, where we feel, “Yes, I just want to be in this world and share the experiences of the characters, based on recognition and aesthetic and ethical appreciation.” Another kind is the critically fascinated, where we read in the moods and modes of a critical audience, wondering why these books are written the way they are and not totally liking the text (but neither disliking it, though still affective). A third kind is based on how (neurotypical) critics have responded to autism/autistic-fiction, investigating what words are used and how they create value. We use the literature to carve out our ways through an autistic readership, centering our reader feelings. This paper should not be read primarily as a paper analyzing the fiction it deals with but as an investigation of autistic reader practices and experiences. Still, the literature infects the reading—the reading cannot be separated from the read texts.

In this paper, we include the following among our readings of autism/autistic fiction:

- Fiction with explicit autistic characters, which can be referred to as “named” representations of autism (cf. McGrath 2017)
- Fictive characters that we read as “moving autistically” or that have been discussed in the paratexts of the books as autistic, regardless of the author’s intention. This is what Mullis refers to as “Autistic coding” (Mullis 2019; see also McDermott 2022). This includes characters with which “we have long associated traits like lack of eye contact, and difficulty with sarcasm and picking up subtle cues, and love of routine, and restless fidgeting or awkward gait” (Mullis 2019, 152), or as McDermott puts it, “characters who are not explicitly identified as autistic but have attributes associated with autism” (McDermott 2022, 59, fn. 10)
- Author ways of writing that we read (or “code”) as moving autistically in that textual space, regardless of the author’s own (explicitly stated) neurological status.

## Neuroqueer reading practice

Miele Rodas (2018) starts to define an autism poetics in *Autistic Disturbances* by seeking out traits of autism in literary texts. This is a way of seeing the autism as inherent in the texts—or *autism as agent*—and does not focus very much on either the writer’s or novel characters’ possible own neurodivergence,^1^ which can be seen as expressions of *autist as agent*. Focusing on autism as agent and representations of autistic involuntariness, Miele Rodas (2018, 4) recognizes “descriptors of autistic expressive practice” as autistic coded rhetoric, such as autistic silence, ricochet, apostrophe, ejaculation, discretion, and invention. From this, she argues that autistic forms of expression have always been integral to the development of the Western literary canon. Focusing on various works and textual examples, from Andy Warhol’s films to the American Psychiatric Association’s *DSM* (*Diagnostic and Statistical Manual of Mental Disorders*) to Bronte’s *Villanelle* and Defoe’s *Robinson Crusoe*, Miele Rodas interrogates the cultural movements and conditions that have led some instances of autistic-coded rhetoric to be touted as artful, creative, and revolutionary while others have been branded pathological or examined through other deficit forms. She stresses that her descriptors and textual examples are a beginning, not something comprehensive (Miele Rodas 2018). An autism poetics—or, in the words of Yergeau (2018), autistic rhetoric—cannot be generalized.

Moving on from *autism as agent* to *autist as agent*, in this text, we use our bodyminds when we read. We read *autistically*, and we are autistic spectators and commentators of what we read and see. By autistic readings, or to read autistically, we mean that our autistic bodyminds—our way of moving through space—affect our readings. Moving autistically can mean different things. We intend to use autism as a neuroqueer interpretative lens. We are not interested in diagnosing or symptomizing but in founding and finding new tools and strategies to develop our autistic ways of reading and writing. We will refer to the texts we work in and with as *textual spaces*. We move through a textual space autistically. Phenomenologically, we experience reading and writing through our autistic bodyminds. In conventional Western thought, there is the tendency to split the mind and body, with the mind being associated with intellectualism and culture and the body being thought of as uncultured, biological, and primitive. This ignores how reading can be a profoundly somatic experience, evoking the senses in new ways and causing the body to react physically (stimming, giggling, crying, etc.). This is part of what Yergeau (2018, 178) defines as an “interbodily invention,” as they argue that autistic embodiment is constantly moving towards new sites of meaning-making, always encompassing physicality. Yergeau explains interbodily invention like this:

But a more current (and relevant) approach to invention, I think, comes from the work of Bre Garrett, Denise Landrum-Geyer, and Jason Palmeri, all of whom position invention in relation to embodied and multimodal communication. In one such offering, they describe invention as the “process of making connections, rearranging materials (words, images, concepts) in unexpected ways. … [Invention] manifests through the body, for a given body actively participates as an inherent material, alongside other materials, other bodies … in the ever-becoming, ever-shifting engulfment of semiosis.” Here, Garrett and her coauthors exhort that embodied communication is not a site for intervention, as many clinicians would have us believe, but is rather a site of invention. (Yergeau 2018, 181)

Included in our bodyminds are our feelings. We use our feelings as part of our interbodily invention to carry through this exploration. We argue that this goes in line with how Murray, Lesser, and Lawson (2005) define the autistic mind as monotropic—and monotropism as an interest *charged with feeling*. Through writing to each other (ourselves as the imagined audience/reader of what we write, our textual space), we establish a reading-writing method that engages in a collective monotropic flow, what Jackson-Perry et al. (2020, 136) have referred to as a “collective experience of monotropism,” which in turn fosters new insights to the reading process as well as the text written.

In this paper, we read autism/autistic fiction autistically as both “sole autists” and in an “autistic togetherness” (Sinclair 2010). As *sole autists*, we read as autists within our own spaces, as singular autistic bodyminds. Within our *autistic togetherness*, we read as autists within our shared textual space, between autistic bodyminds, as a collective bodymind. Altogether, this results in a sense of a collective autistic “hivemind” moving in the textual space together. At the same time, this movement is being disrupted into singular bits and thoughts, moving in the space in different directions, at different times, and in different moments. It is not linear. It is associative. It is an inductive bottom-up approach (cf. Mantzalas et al. 2022). It is asynchronous communication. It is parallel play and parallel talk. By *parallel*, we refer to being in a conversation where talk and action do not necessarily follow a strictly dialogical structure but sort of work in parallel to create a multitude of meanings. It is an intense sense of togetherness in the collective monotropic flow.

We think of neuroqueer readings not only as a *doing*—the readings from our autistic bodyminds—but also as a *founding*. Through our readings, we found “the concepts in which to think autism” (Hacking 2009a, 1467). Paraphrasing Hacking (2009a), we argue that a neuroqueer reading has an important role in the ongoing social and cultural evolution of neurodivergent and neurotypical ways of reading autism/autistic fiction. In line with his idea of looping, Hacking argues from his readings of autistic autobiographies:

The story-tellers learn from autobiographies how to tell their tales. But that is a two-way street. Temple Grandin’s *Emergence* was written before the genre got underway, so her self-descriptions are unaffected. Today’s autistic child, brought up on children’s stories about autistic children, and who in later years goes on to write an autobiography, will give accounts that are textured by the early exposure to role models. (Hacking 2009a, 1469)

We do not agree with Hacking’s depiction of Grandin’s storytelling and her self-descriptions as “unaffected.” Rather, we think of her storytelling as affected differently than after “the genre got underway.” Our accounts are similarly “textured by the early exposure to role models.” When we are reading *Animals in Translation* by Temple Grandin (2005), we think about the closeness between Grandin and the animals and what the closeness between Grandin and the animals *does*. But we also start to think about the impact of Grandin’s co-author, Catherine Johnson. Johnson is described in the author’s description at the end of the book as a non-autistic mother of an autistic child and a psychiatrist. Reading *Animals in Translation*, we cannot know for sure who is the author and who is doing the editing on whose narrative.

While Hacking is getting at the same thing, saying that how we read and interpret has real-life resonances for autistic people (and non-autistic people’s views on autistic people), he is looking at autism/autistic fiction with a neurotypical gaze. We think it is a shame that people write about autism badly. By this, we mean interpreting or translating autistic experience/autistic ways of being from a deficit lens. This includes looking at autism and autistic ways of being from a neurotypical gaze where autism is being negatively contrasted to neurotypical experience/ways of being, what Walker (2021) has referred to as the “pathology paradigm” of Autism. Looking at autism (and other forms of neurodivergence) from a neurotypical gaze does not only involve centering deficits and contrasts to neurotypicality, thus constructing neurodivergence and neurodivergent people as objects of treatment, “saving,” fetishisms, and belittling; these bad writings and interpretations of autistic ways of being make autistics suffer. Instead, we suggest, a neuroqueer reading put us in the driver’s seat in the level of reading, writing, and interpretation.

In the following sections, we set out the contours of our neuroqueer reading/writing practices, including:

1. Neuroqueer phenomenological readings: Moving autistically in that textual space.
2. Joint action: Neurodivergent collectivism.

## Neurotypical readings of autism/autistic fiction

Some writers have illuminated aspects of the autistic triad of social impairment, abnormalities of language, and need for sameness. Other writers have opened *our* eyes to *the autistic worldview* in its *strangeness and richness*. Still, more have started to examine prejudice, disability rights and the implications of an international autism community. (Bates 2010, 47; our italics)

In recent years, autism/autistic fiction has been increasingly produced in literature, cinema, and TV. Cultural attitudes may be changing as autistic “strengths” are becoming more widely portrayed; however, there is still a fundamental issue: these seemingly positive portrayals are governed by neurotypical readers’ attitudes, expectations, and opinions about what autism *is* or *means*. Autistic characters too often feature as “prosthetic devices” (Mitchell and Snyder 2000, 3), narrative vehicles to explore neurotypical issues and concerns, tragic heroes, or objects of pity. Reading Bates’s (2010) readings of autistic autobiographies, we start to ponder the “our” and the positioning of “the autistic worldview” as “strangeness and richness.”

Autism/autistic fiction, or what Ian Hacking (2009a, 1468) refers to as “Autism stories,” is creating a language of autism, where previously “there used to be no language in which autistic experience could be described” (Hacking 2009b, 499). This, Hacking argues, “affects how autistic people think of themselves. It certainly affects how nonautistic, ‘neurotypical,’ individuals think about autism. Not all of this is a good thing, for all too many stories foster images of ‘the’ autistic person as having special gifts that ordinary people lack” (Hacking 2009b, 499). The cautionary tale about representing autistic people as “having special gifts that ordinary people lack”—as being extraordinary in contrast to neurotypical ordinariness (cf. Garland Thomson 1997)—mirrors a common literary trope of autism, which Murray (2006, 27) has referred to as “the sentimental savant” (see also Moore 2019). Similar to Bates’s (2010, 47) noting that autistic “writers have opened our eyes to the autistic world view in its strangeness and richness,” Siegelman describes *The Curious Incident of the Dog in the Night-Time*, by Haddon (2003), as a novel that “*remarkably* captures the inner world of an adolescent boy with Asperger’s syndrome, characterized by autistic hallmarks of social aversiveness and susceptibility to stimulus overload but with remarkable development in a single area, in this case, mathematical genius” (Siegelman 2005, 57; our italics).

Among the paratextual material of *A Room Called Earth*, by Ryan (2020), reviewers are similarly praising the book for offering something “remarkable” and “unique” but also “humane.” A reviewer writes: “A Room Called Earth offers a *strikingly unique* look at intimacy, identity, and time itself. From now on, I want every novel to be this fiercely autistic, this assured, this untethered from the status quo. Madeleine Ryan is a wholly original writer; this debut announces a tremendous talent” (our italics). Another reviewer writes:

In prose filled with humor and warm light, Madeleine Ryan unearths the bright, luminous soul of each animate and inanimate being she encounters. Instead, *remarkably*, it is the self shaped by and against social norms that is met as other. The result is an intelligence that feels not only totally refreshing and original but *wonderfully humane*. (Our italics)

In another reading of *The Curious Incident of the Dog in the Night-Time*, Flajšarová (2018, 43) associates the novel with an expression of a “fundamental change in the perception of modernism,” as a “postmodern form of a disrupted text.” Thus, traits of autism could be read as part of a postmodern aesthetics—in the same way as Rodas (2018) characterizes the Romantic period as quite autistic.

Reading autistic ways of writing, autistic characters, and autistic narratives as something *Other*, as part of a literary trend, or as part of specific genres makes visible how traits of autism can work as artistic tools; being autistic is being artistic. At the same time, positioning autism and the autist as a neurological Other, thus reducing autism to a literary trope, risks reducing the biographical and existential experience of living in an autistic bodymind. Being autistic readers of autism/autistic fiction, we learn to be grateful for the fact that we exist. And we are grateful for representation. But the representation comes with a new kind of violence: the violence of a reductive understanding, the neurotypical understanding of the autistic deemed as always more valid and objective than the autistic person’s own. Yergeau (2018, 2) notes, “Even when autism is depicted as a condition that resists the narratable … the narrating impulse remains entrenched in the act of diagnosing unto itself: Traits and check boxes tell a story.” In the following extracts from our autoethnographic writings about our reading experiences, we reflect upon this neurotypical storying of autism and the autist.

I always ask myself, when autism is theorised in terms of a postmodern aesthetic, or through any other neurotypical interpretative lens or framework, who is this being written for? Is it neurodivergent-led and/or does it reflect the desires of neurodivergent communities? I usually find that the answer is “probably not,” as it tends to pander to current trends in literature, about how autism “represents” the postmodern condition or how it provides “insights” which “enrich” the ways we read and write. Even when these are well-meaning (see, for example, essays in *Autism and Representation*, edited by Osteen 2008), they tend to miss the mark completely. I’m not saying this is the case with all of them, and I don’t think this is what Rodas (2018) is doing, but I wonder how much literary theory as a discipline actually cares about neurodiversity? Something we can maybe talk about. (One of us)

I think one of the problems with characterizing autism as postmodern (or any other kind of ism or trend) is that it makes it possible for autism to be trendy, which means it can also be un-trendy. Autism is not fixed in time—even though some cultural historians may argue that it is, and the diagnosis might be, but the autistic being is not. Reducing autism to an aesthetic is also problematic since it makes the autistic subject invisible, reducing them to an experience, an event, that should be open for all (neurotypicals) to take part in. The thing with working in a separatist autistic (reading) community is that it creates a space where autism is ordinary, which makes it possible to investigate the nuances of autism that colors every individual—in contrast with the neuromixed and neurotypically dominated space, where autism is always reduced to a single experience, the same for all autistics. Just like a neurotypical experience is inaccessible to the autistic, the autistic experience is inaccessible to the neurotypical (cf. Milton 2012), and even though it is a good idea to try to understand each other, the best understanding is probably to understand that one does not understand—a part of understanding that neurotypicals seldom get. (One of us)

### Neuroqueer phenomenological readings: Moving autistically in that textual space

*Dragon Pearl*, by autistic author Lee (2019), revolves around a group of characters with extraordinary abilities—supernatural creatures. The supernaturals stem mainly from Korean mythology: foxes, tigers, dragons, goblins, ghosts, trolls, and fairies. We read them as metaphors for characters differently positioned on a neurological spectrum. All supernaturals have different (equivalent to neurodivergent) superpowers. Among them are the ability to transform into something one is not (or what has been referred to as “autistic passing” [cf. Pearson and Rose 2021]), increased sensory sensitivity, and the ability to feel other people’s feelings or sense if a person is telling the truth (or what Shore [2003] has referred to as “fusing”). In line with the importance of pointing out the breadth of what *autistic-ness* can be, Lee points out in *Dragon Pearl* the individuality of the supernaturals. The main protagonist of the novel, Min, is among the supernaturals. In an exchange, Min’s brother, who has become a ghost, tells Min: “Every ghost is a different person. Sometimes we want different things, too. I still want to visit every one of the Thousand Worlds” (Lee 2019, 295–96). “There are millions of ways of being autistic. There are as many worlds as there are autistics” (One of us).

Following the ideas of autistic fusing as developed by Stephen Shore (2003), Ralph Savarese argues, from his explorations of autistic reader practices, that autistic readers easily are “‘fusing’ with another’s suffering, whether real or imagined” in their readings (Savarese 2018, 18). Developing the idea of autistic reading as an experience of fusing, we start to think about autistic reading as an act of sharing experiences with an author and literary characters of the world. We started to think about autistic reading and writing as “autistic acts of love and care” (McDermott 2022, 59). From the perspective of sharing experiences, movements in the (literary) world can be seen as love and acts of love. The movements in the world can be illustrated by our reading of *Movement: A Short Story about Autism in the Future*, by Fulda (2012). *Movement* is a novel centered around the autistic, nonverbal dancer Hannah and her non-autistic family. Fulda, herself a non-autistic mother of an autistic child, writes in the novel about Hannah’s love of her ballet shoes as an extension of her body, movement at the moment, loving the things, loving the world. Unlike many novels picturing autistic characters from a non-autistic outsider perspective, the picturing in *Movement* is a careful outsider interpretation or translation, openly admitting the novel as an interpretation from an outsider’s perspective. What happens in *Movement*, in contrast to what we experience when reading Grandin and Johnson’s *Animals in Translation*, is that Fulda does not try to explain or claim that she fully understands; she puts herself close to, nearby, yet still acknowledges the (though small) distance. A translation can never claim to fully translate the work it translates; there will always be a slight shift. This shift, we argue, is not hidden in *Movement*, nor is it put into a neurotypical frame. Hannah’s love for her shoes is not psychologized in the way Grandin’s understanding of the horses in *Animals in Translation* is. There is, in *Movement*, no explaining through established neurotypical theories; rather, the mother’s closeness to her child seems to be enough to grasp a kind of empathy without complete understanding.

Similarly, we argue, autistic reading can be seen as autistic moving in textual spaces, as a loving and careful sharing of experiences in the world, sharing a moment of intense (monotropic) feeling. Sharing movement(s) at the moment(s) in the world is an invitation to fuse; it includes sharing experiences as pictured in novels (through the rhythm, language play, form, and textuality of the text in itself) and also the experiences of main protagonists who may not be explicitly positioned as autistic but who, from our reading, move through a space autistically. This movement can mean different things. Elsewhere, one of us (Anna) has written about Fagerholm’s (1998) novel *DIVA* (see Nygren 2022). The novel’s main female protagonist, Diva, is not an explicitly autistic character; she has not been diagnosed as autistic, but the character, as well as other characters in the novel, and the way of the author’s writing can be read as moving autistically.

Fagerholm’s alliance with the girl Diva is complete, and I read the traits of autism in the novel as small gifts to me. It is about a young girl, with a colorful sexuality, complex friends and family, and a nerdy and overwhelming interest for Western philosophy. *DIVA*, similar to *Dragon Pearl* by Lee (2019), criticizes the narrow human civilization by making it funny, by focusing on the small dogs, and by becoming a butterfly. The act of reading the autism where it is not already written is an act of taking back the narrative, and the language. I am an autistic reader, reading autistically. Reading autism/autistic fiction sometimes feels like reading [the] *DSM*-5 (APA 2013). For the neurotypical audience. Reading neurotypical (?) fiction with an autistic gaze, and doing my diagnostic work, helps me understand how it works to move autistically in a (textual) world. The pleasure of calling you autistic. Of pointing out the traits of similarity between you and me. Of saying we are, like Diva, one-of-a-kind, but also almighty. My world is autistic, that won’t stop my anger but feed it. (One of us)

In the intergalactic future world of *Dragon Pearl*, the supernatural characters must constantly pass, living at a constant risk of being exposed. Min is a fox and has grown up in a fox family, which we read as a neurodivergent family. In her immediate extended family (mother, siblings, aunts, and cousins), the importance of blending into the environment is emphasized, but here other fox relatives have rather chosen to take advantage of their abilities and made a fortune by deceiving “ordinary people.” The events in the book start when the fox family is visited by an outside (non-supernatural) detective who is to investigate Min’s brother’s disappearance/possible desertion from his service in the military to find the mysterious artifact Dragon Pearl (which is some kind of holy grail). In the novel, other characters’ intentions and feelings are read through smell, which we read as an illustration of autistic fusing.

The investigator looked down at me. If I’d been in fox shape, my ears would have flattened against my skull. His expression wasn’t condescending, as I would have expected. Instead, I could sense him measuring me. And now I could smell some suspicion coming off him. Did he think I was hiding something? (Lee 2019, 9)

What should I do with the smells of love/life (or in Swedish: kär/leken (kärlek=love, lek=play)? It’s so hard to come close to others because I need to accept the smells and almost all of them are intruding. I feel like an animal. Temple Grandin (2006) writes in her book *Animals in Translation* about being autistic and close to animals. Like a special connection. I get that it is problematic (as if autistics were more like animals than humans), but I can only accept the smell of Zlatan (I mean my cat Zlatan, I would never smell Zlatan the human football player).

I think about the fact that it is a fox—as sly as a fox—the animal who is a metaphor for slyness (being foxy)—in Swedish it is: “listig som en räv.” The word “listig” contains the word “list.” And I think about listing as a typically autistic thing—how Miele Rodas writes about it in *Autistic Disturbances* (2018), I think about defeaturing “listig” into “listing,” I think about the fox searching for traits of autism in the text. I think about the Risk. What do I risk when I say I am autistic? I risk being reduced to symptoms of my diagnosis and therefore something not even worth thinking about. (One of us)

There is a difference between authors sharing experiences with the reader and authors explaining things to the reader. In the novels by Lee (2019, 2020a, 2020b), in *DIVA* by Fagerholm (1998), and in *Movement* by Fulda, as well as in the novel *A Room Called Earth* by Ryan (2020), the authors rather share experiences with the reader through (more or less thick) descriptions of sensory experiences. The writing becomes an invitation from the author to the reader to play and share experiences with the author, to point out the traits of similarity between the reader/you and the author/me. In contrast to these authors, who are *talking*/sharing an experience *with* the reader, as in the invitation to share movements and moments in the world, in other instances, the author is rather *talking*/explaining things *to* the reader. This is illustrated by our readings of *The Grasmere Cottage Mystery* by autistic author Dahlia Donovan:

Sitting back on the carpet, Valor rested his hands lightly on Bishan’s sock-covered feet. *His Bish* hated wearing shoes; *it was always a struggle to get him* put anything on his feet even during frigidly cold winters. He claimed anything aside from fabric restricted his toes too much. Valor chalked it up to another way being autistic made Bishan uniquely perfect. (Donovan 2018, 23; our italics)

“That’s a dead body in our garden.” He risked a glance at his long-time boyfriend to find him mesmerized by the sight. “I mean, it’s a corpse.”

“Very astute of you. Very. Astute. Quite astute. Incredibly so, actually.” Bishan had a tendency to repeat words when he enjoyed the way they sounded to him. He claimed it was one of his many autistic superpowers. “Ahhh—stute.”

“Yes, I grasp the concept. Why’s a body in our rose bushes?” Valor carefully set the mug into the sudsy water, drying his hands off on his jeans and ignoring the indignant huff from Bishan. “Right. I’ll call the police, and you put Staccato in the bedroom.” (Donovan 2018, 3)

*The Grasmere Cottage Mystery* is centered around a neuromixed romantic relationship between autistic Bish and non-autistic Valor. In the novel, the author most often talks *to* the reader and explains *to* the reader, as a God’s eye or an all-knowing author. Both the talking/sharing with and the talking/explaining to can be seen as ways of role modeling: roles for both autistic people and non-autistic people, how to move in the world, and how to cross-neurotype interact.

I know when I read *Dragon Pearl* that it is Lee’s “fault” that I feel so loved by the author. There is no explanation for an extraordinary incomprehensibility, that would make me feel estranged as a reader. There is just a giggling, happy, sometimes scary-sweet thing, and we started to play. Reading Donovan, I feel like a stranger. It is like I, with the book’s autistic character Bishan, keep playing with “Ahhh—stute.” When Valor/the real reader/the author says: “Yes, I grasp the concept.” Silence. If you want to keep on reading, be quiet.

Aaaaaaahhhhhh. Oh, (Swedish word plays, gets to my mind, I recognize myself in that, literal translation: I do feel again me—I do feel me again—when I say aaaaaaaaaaaaaaaaaaaaa, I do feel myself again, I let myself keep on languageing; stute, stutt, stutter, stamma, stem… If someone says: I get it. Then I would be quiet. It is a kind of goodhearted understanding, that really stops the flow. Stut-stud-studera-deras-era-agera-bageerah-geeh-aaaaaaaaaaaaahhhh. Is the neurotypical understanding the hardest kind of goodness? Because you’re expected to be happy. As if it’s expected of you to smile when they get it. Thanks. Yes. (One of us)

### Joint action: Neurodivergent collectivism

Sharing movements and moments in the world can be interpreted as an invitation to “joint action” (Francis and Hey 2009, 231), suggesting different ways of neurodivergent collectivism. Sometimes this is expressed in loving acts towards different Others (regardless of their form or functionality), sometimes in a general loving of the world. In several novels by Yoon Ha Lee, joint action is done between a diverse range of characters positioned as Others in contrast to dominating groups of humans: machine Others, supernatural Others, ethnic Others, and gendered Others. In *Phoenix Extravagant,* the novel’s main protagonist, Gyen Jebi, the non-binary artist from a colonized ethnic group, is animating a machine Other through recognition and translation/support to communication (Lee 2020b). In Lee’s short novel *Beyond the Dragon’s Gate*, the protagonist, the researcher Anna, asks her superior, the Marshal: “Do you ever treat your ships the way you would your lowliest soldiers?” (Lee 2020a). Similarly, in *Dragon Pearl*, Min notes:

I couldn’t shake the feeling that the battered freighter was eager to make the jump. In the old stories, older even than the Thousand Worlds, a humble carp could become a dragon by leaping up a waterfall. If a fish could dream of upgrading, I didn’t see why a starship couldn’t, in its secret crystal heart, have ambitions, too. (Lee 2019, 72)

In several of Lee’s novels, machines are commonly alive but live their lives as unrecognized and silenced members of a starship crew or groups of the military. In all novels, the othered protagonists are supporting machine Others to become empowered selves through understanding in ways other humans cannot—to lovingly act towards them as “they’re people, too” (Lee 2020a). The protagonist Min in *Dragon Pearl* is also appreciated based on her various abilities and personality. In addition to her sensory sensitivity, she has technical talents that are useful in several dramatic situations in the book, including the ability to enter into a monotropic interest-driven strong focus and, with it, an ability to connect mentally with machine Others. The spaceship she is on is given some form of her consciousness in the book, even if this is not expressed through explicit verbal communication. She is, however, represented as someone worthy of love and who loves mostly based on who she is, not on what she does.

I have problems reading Lee’s work. They stress me because there are always a lot of troubles that the character [Min] needs to solve. Everything is a problem. It is a perfect depiction of the social model of disability: The disability is not within a disabled person, but the disability lies within the surroundings. Lee’s characters are perfectly understandable—but the world is so stressful! I really get why you [Hanna] love their novels, but to me, there is too much stress… (One of us)

In the next section, the joint action is illustrated by solidarity between neurodivergent others, in worlds explicitly aiming at neurodivergent extinction/adaptation of the neurodivergent mind, where the neurodivergent community functions as a kind of resistance—through joint action or neurodivergent collectivism—to humanness that does not really embrace us. In the novels by Lee, but also in *A Kind of Spark*, by autistic author McNicoll (2020), neurodivergent experience is portrayed both in spaces dominated by neurotypicals and in spaces dominated by other neurodivergent people. Here, the neurodivergent character is never passive but is portrayed as someone who, with their agency, navigates in various spaces and is multifaceted, not necessarily evil or good. Recurring is a form of group solidarity, where you support each other in different ways and where the support is mutual rather than one-sided—for example, when the autistic big sister Keedie in *A Kind of Spark* says to her autistic little sister Addie:

I open my mouth to say something more but Keedie places a hand on my arm. “Don’t, Addie. He wants to believe his small-minded nonsense, let him.”

“But I don’t understand.”

“When grown-ups don’t like what we have to say, they blame our autism and say we don’t know our own minds.” She exhales and shrugs. “It was textbook when I was at that rubbish school. Constantly accused of copying. They couldn’t believe the ideas were mine.” (McNicoll 2020, 44–45)

We read Lee’s novels as a critique of the narrowness of what is being included as human, or what can be seen as failures of performing “typical human emotions and interactions,” positioning the subject at risk of “failing to qualify for the ‘human club,’ and instead is seen as something other than human” (Bergenmar, Bertilsdotter Rosqvist, and Lönngren 2015, 215). In *Dragon Pearl,* the neurotypical gaze comes in the shape of an ordinary human gaze, more or less explicitly visible as such. From this human gaze, all biological Others are lumped together as supernatural creatures and are expected to pass, accepted “as long as they confined themselves to human form” (Lee 2019, 91–92). At the same time, a distinction within the group is made between the more respectable and the less.

The Space Forces accepted the “more respectable” supernatural creatures, such as dragons and celestial maidens—and even tigers if they could control their violent tempers—as long as they confined themselves to human form. Dragons, in particular, were enormous in their true manifestations. It was easier to design starships for human shapes and sizes and have everyone else adapt. (Lee 2019, 91–92)

“What should one do with the (non-)acceptance? And the fact that *some* are respectable and others are not? A first and second-class deviation. I frown. On the verge of being a little human” (One of us).

## Conclusions

In the essay “The Foreign Gaze,” Herta Müller (2009) writes from a position in exile about what she calls “the foreign gaze” that occurs among those who live in a nation where persecution is common. What is interesting about Müller’s take on the Foreign and the gaze is that the gaze belongs to the exiled or persecuted; it is not the majority view *of* the minority or vulnerable. Müller’s agency is therefore put with those who otherwise are often called *Foreign*. To live with persecution means, according to Müller, to lose trust in daily objects, because all things might carry surveillance and judging. The exile and the autism are not the same things, but there is a similarity. The Foreign is created within the autist when they meet a world that does not confirm their view of the world.

You learn, as an autist, that the world as you see it, does not count, and you’re forced into a position where you start to estrange yourself. I learn at the hospital that others know more about me than I do. As if the doctors and psychiatrists surveil me as a totalitarian state. I think I need to learn to love the foreign, to cope with myself and the world. (One of us)

Yergeau (2019, 10) argues: “It’s important to note that whatever the placeholder—whether schizophrenia, autism, depression, cerebral palsy, ADHD, bipolar—mental disability signals a kind of rhetorical involuntariness. Mental disability wields more agency than mentally disabled people.”

Being rhetorically involuntary, does that also includes being involuntarily rhetoric? I sometimes feel [that] saying I’m autistic has the effect of a war machine. It creates a silence in people, a silence broken by the compliment “But you don’t look autistic,” a line that silences me, in anger, a silence broken by the neurotypical person telling about someone they know with autism, and how well they have learned (been taught) to function in society. My diagnosis and my autistic being, the autistic statement, seems to force neurotypical people to tell that they KNOW about autism. The word AUTISTIC is immediately turned into AUTISM, making me a part of the disability, and yet, distancing me from other disabled. (One of us; capitals in original)

Moving autistically through a textual space sometimes pushes us into this involuntary rhetoric, where autism is a dramaturgical function. Reading this function as an autistic reader creates a feeling:

I move through the text blaming myself for being 1) autistic in the cliché-way that is told, 2) not being autistic in the correct way that is being told, 3) satisfied with being tolerated, 4) not being satisfied with being tolerated, 5) in love with the small parts I recognize within myself (how egocentric is it possible to be?). Until I find these few golden pieces of autistic literature that works autistically in my brain. (One of us)

We think of movement as both a work of cleaning and to be done with the cleaning. Through moving, we are cleaning away all the garbage that is in the neurotypical prejudices and well-meaning understandings of the neurotypical teacher/parent/doctor/colleague (cf. Botha 2021a). Botha (2021a) and Pearson (2021) have both pointed out that autistic people are often the ones doing all of the heavy lifting to make autism research more ethical, questioning poor theory and shoddy methodology, what we have referred to as bad writings. However, the collective way of being autistically in a text feels like being temporarily done with the cleaning; it feels like stepping out in the sun (literally the same feeling of warmth and light and being almost dazzled by the light). Yergeau notes:

[Autism] represents the edges and boundaries of humanity, a queerly crip kind of isolationism. We, the autistic, are a peopleless people. We embody not a counter-rhetoric but an anti-rhetoric, a kind of being and moving that exists tragically at the folds of involuntary automation. … Under such logics, I have written this book, presumably unaware of my reader and my (non)self. The involuntary actions, thoughts, writings, and behaviors of my autistic body negate my claims to writerhood, rhetorichood, and narrativehood. Instead, this book might be better understood as a cluster of symptoms. (Yergeau 2018, 11–13)

We want to move towards collectivism, as authors collaborate to form a *hivemind*, a people (rather than staying as “peopleless people,” as Yergeau puts it). We wonder how much of this *individual versus collective voice* is a theme in autism/autistic fiction and self-advocacy literature:

“We” are never fully allowed to be a collective “us.”

I have been thinking a lot in my research about how autistic authors (most often memoirists) use the collective “we” and “us.” How does the collective voice appear in our reading practices? Relatedly, there is the differentiation between reading as “sole autists” and reading with a sense of “autistic togetherness.” It makes me wonder more about the origins of a voice, especially within activist movements, where we’re supposed to be fighting for the same thing. I don’t have much to say about it, except that it’s a thought I keep returning to. (One of us)

Through reading together and sharing our reading through reading diaries, we get into a textual collective autistic flow. Being autistic together means being unique—since there are millions of ways of being autistic—but it also means not being unique. We do not agree with the use of *unique* in the way neurotypical reviewers have referred to Ryan’s *A Room Called Earth*—that is, as a way to position the autistic character of an autistic author as an isolated person and experience. Sharing an interest charged with feeling means feeling together. In this paper, we have worked through a collective feeling-writing method. We read with our thoughts and feelings. Doing this together is a way of recognizing each other’s experiences of texts. Reading autist-written fiction as an autist is not always easy or pleasant. We have found internalized diminishing behaviors among autistic characters and authors—we have felt sad about this. Recognizing this sadness as a valid response to a text that presumably has as its goal to be empowering is a way to take seriously different ways of being autistic and being activistic. We want to see a deepening discussion within and between autistic communities about how textual practices shape our ways of seeing ourselves.

We have, in this article, developed one neurodivergent approach (of many possible approaches) to literature—foremost but not exclusively focusing on fiction reading. We have used a method of reading where the fictional works influence the theoretical field in a way similar to Toril Moi’s. Moi (2017) argues for a philosophical reading, where theoretical works and fictional works are parallel read, and the fictional works are read as much as a philosophical statement as the theory. We add the layer of the reader experience, giving as much attention to our reader texts as to the fictional and the theoretical texts. We have written from a personal view and taken our own autistic experiences seriously. We have tried to carve out one neurodivergent critical view (or a few of many neurodivergent critical views) on autism/autistic fiction. We have allowed ourselves to be critical of fiction written by autistic authors—when we feel that the autistic subject/agent is still objectified as something strange in need of explanations. We have found joy in the small sparkling pieces. We have found love in our reading bodyminds. Working collectively has allowed us to feel in multiple ways—it has been a work *charged with feeling*. We have allowed ourselves to be emotionally academics and to have academic emotions.

## Endnotes

^1^Bertilsdotter Rosqvist, Stenning, and Chown define and discuss the term “neurodivergence/neurodiversity” in the introduction to the anthology Neurodiversity Studies. A New Critical Paradigm as follows:

The concept of neurodiversity usually refers to perceived variations seen in cognitive, affectual, and sensory functioning differing from the majority of the general population or “predominant neurotype”, more usually known as the “neurotypical” population. The ontological assumption of neurodiversity is often contrasted with the ideological position that there is a recurrent “normal” cognitive, affective, and sensory type, otherwise known as the normal human being or the “normate” (Garland Thomson, 1997), as defined in the first half of the twentieth century, by way of the prerogative of psychologists who were living in the shadow of eugenics. The attribute of neurodivergence may also be applied—more problematically—to individual people. As Nick Walker says, “Diversity is a trait possessed by a group, not an individual” (2014) and to talk of individuals as neurodiverse is to situate them as “other” to the norm (as well as being, in our opinion, nonsensical). (Bertilsdotter Rosqvist, Stenning, and Chown 2020, 1).
